# The *g3mclass* is a practical software for multiclass classification on biomarkers

**DOI:** 10.1038/s41598-022-23438-9

**Published:** 2022-11-05

**Authors:** Marina A. Guvakova, Serguei Sokol

**Affiliations:** 1 Department of Surgery, Division of Endocrine & Oncologic Surgery, Harrison Department of Surgical Research, Perelman School of Medicine, University of Pennsylvania, 416 Hill Pavilion, 380S University Avenue, Philadelphia, PA 19104 USA; 2 CNRS, INRAE, INSA, Toulouse Biotechnology Institute, University of Toulouse, 31077 Toulouse, France

**Keywords:** Diagnostic markers, Software

## Abstract

The analytes qualified as biomarkers are potent tools to diagnose various diseases, monitor therapy responses, and design therapeutic interventions. The early assessment of the diverseness of human disease is essential for the speedy and cost-efficient implementation of personalized medicine. We developed *g3mclass*, the Gaussian mixture modeling software for molecular assay data classification. This software automates the validated multiclass classifier applicable to single analyte tests and multiplexing assays. The *g3mclass* achieves automation using the original semi-constrained expectation–maximization (EM) algorithm that allows inference from the test, control, and query data that human experts cannot interpret. In this study, we used real-world clinical data and gene expression datasets (*ERBB2, ESR1, PGR*) to provide examples of how *g3mclass* may help overcome the problems of over-/underdiagnosis and equivocal results in diagnostic tests for breast cancer. We showed the *g3mclass* output’s accuracy, robustness, scalability, and interpretability. The user-friendly interface and free dissemination of this multi-platform software aim to ease its use by research laboratories, biomedical pharma, companion diagnostic developers, and healthcare regulators. Furthermore, the *g3mclass* automatic extracting information through probabilistic modeling is adaptable for blending with machine learning and artificial intelligence.

## Introduction

Targeted therapy is a crucial focus of drug development and a cornerstone of precision medicine^1^. However, molecularly targeted therapies and immunotherapies are expected to benefit only a subset of treated patients. Identifying individuals likely to benefit from the targeted treatments and monitoring resistance to new cancer therapies depends heavily on biomarkers. This broad category of analytes, including biochemical, genomic, and proteomic measurements, may indicate an underlying disease mechanism or predict a response to a drug^2^.

With the rapid adoption of high-throughput assay technologies, discovering “promising/potential/candidate” biomarkers and therapeutic targets has exploded, producing an enormous volume of raw data needing human and robotic intelligence assessment^3,4^. To discover biomarkers, researchers often start by comparing references (a healthy population) and tests (a sick population), assuming the normal distribution of measurements in each group. However, this assumption is frequently not tenable as the real-world readouts of laboratory tests rarely fit into one normal (Gaussian) distribution^5,6^. The best-known example of such biomarker in oncology is a human epidermal growth factor receptor 2 (HER2) overexpressed in a fraction of breast (15–30%), gastric and gastroesophageal (10–30%), ovarian (20–25%), endometrial (14–80%), bladder (23–80%), and lung (up to 20%) cancers^7,8^. Furthermore, two distributions of continuous values from test and reference are rarely fully separated^9^. The overlapping distributions create a methodological dilemma for choosing a diagnostic cutoff value that impacts the diagnostic accuracy and clinical decisions^10^. Although an arbitrary cutoff between the positive and negative results remains a routine clinical practice, there is uncertainty (and hence concern) that dichotomization on biomarkers reflects the continuous clinical risk from a measured test value^11^. Overdiagnosis may cause unnecessary morbidity and cost, whereas underdiagnosis may increase the risk of disease progression. Innovative statistical approaches that can address these deficiencies are needed.

The probability approach is a statistical concept that measures the likelihood of something happening. It has been exploited successfully to analyze the underlying complex structure of text documents, image objects, and voice signals in many subject areas, including biology and medicine^12,13^. Gaussian Mixture Model (GMM) is the flexible probabilistic approach that can characterize multimodally distributed continuous variables^14^. Each class is modeled according to a different Gaussian distribution. In addition, Bayesian classifiers can be used to predict class membership probabilities, such as the probability that a given value belongs to a particular class. The diagnostic applications of Bayesian analysis in medicine have been evolving over the past few decades^15–17^. We recently introduced the method for multiclass cancer classification on biomarkers by applying a Bayesian approach^18,19^. The proposed method allows the discovery of previously unknown groups with different levels of biomarkers by extracting information from molecular assay data using probabilistic modeling. The performance of this method has been validated over datasets of more than 300 clinical samples. In addition, it has been shown to improve the binary classification of clinical markers (HER2 and steroid hormone receptors).

Given these successes and the need to improve disease diagnosis and personalized treatment options, we developed *g3mclass*, a practical Gaussian mixture modeling software for molecular assay data classification. It is intended for tests where the random variation of the parameter-of-interest is an essential component of the modeled situation. In this article, we introduce and employ *g3mclass* using a real-world example of human breast tissues. Samples were classified on clinical markers to help distinguish patients most likely to benefit from the Food and Drug Administration (FDA)-approved targeted therapies while sparing others that need different treatment.

## Results

### Overview of the *g3mclass’s* functionality: or how it works

**Figure 1:**
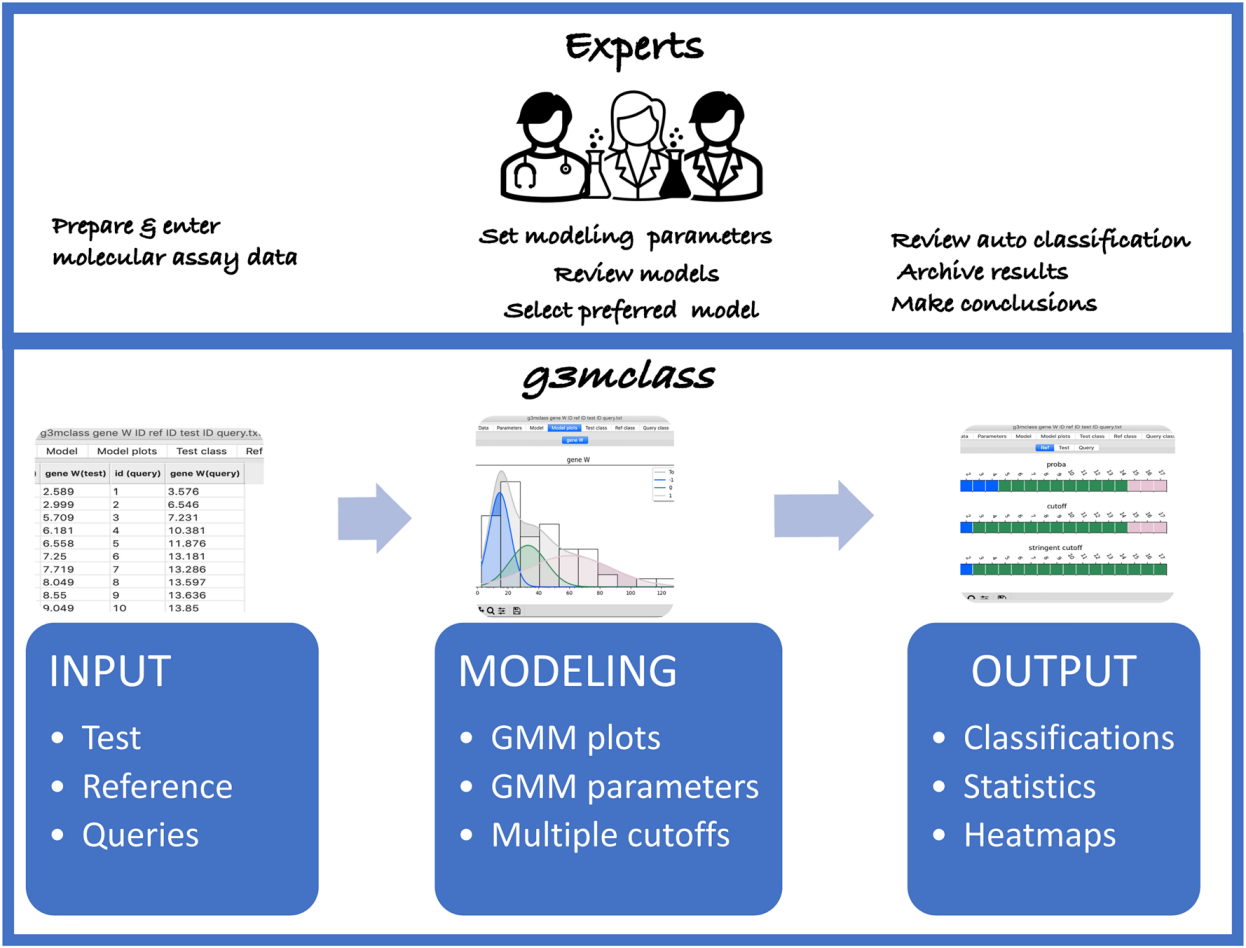
Graphical abstract of the *g3mclass* workflow.

*The g3mclass* is a probabilistic modeling-based classification and visualization software purpose-built for analyzing laboratory assay data. Our development of *g3mclass* was motivated by the outstanding problem of classifying test results in the research laboratories and clinical settings, considering the prior knowledge of the reference. In this regard, Bayesian statistical methods to update pre-existing information about the likelihood of the change provided a robust system for our development of the *g3mclass*. The *g3mclass* core function requires two kinds of data entries for each analyte: test (e.g., disease, treated) and reference (e.g., healthy control, nontreated). Additionally, we built a complementary capability in this software to classify new data (e.g., suspected disease) obtained by the same assay but from an independent source. These incoming unknown data are queries. Thus, the *g3mclass* workflow includes data preparation and input, probabilistic modeling, automated classification of the test, reference, and optional queries, followed by the analysis and archiving of the results (Fig. 1).

**Figure 2:**
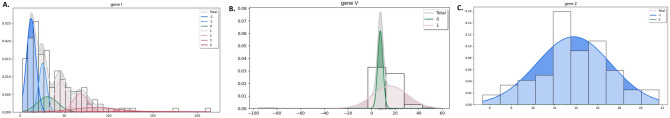
Examples of types of the *g3mclass* created model plots.

The *g3mclass* learns the total test GMM and separates modes composing the mixture upon data input. The GMM learning is based on our semi-constrained algorithm, a modification of the original expectation–maximization (EM) algorithm^20^. To detect test classes distinct from the reference, we constrained the position (i.e., the mean value), spread (i.e., the standard deviation value), but not the weight of the test class 0 to be equal to the corresponding reference values. All other test classes have all parameters adjustable. While our semi-constrained approach preserves parameters of the reference class, it does not affect the convergence and high speed of the analysis regardless of the overlap between the reference and the test values. For example, using a laptop computer, the *g3mclass* runtime for classifying ten analytes with thousands of measurements takes less than 20 s. In the test classes labeled with negative (e.g., − 1, − 2, etc.) and positive (e.g., 1, 2, etc.) integers, the mean values are lower and higher than class 0. The greater the absolute integer value, the further the class is positioned relative to class 0, as illustrated in a plot created by the *g3mclass* software (Fig. 2A). To learn the test model, the software initializes the semi-constrained EM algorithm with classes corresponding to peaks in the histogram calculated on the test sample. The software depicts the model as a probability density function (PDF) overlaid on a test histogram. Because information about the number of bins and their boundaries is not inherent to the data but will influence a GMM, *g3mclass* can vary binning parameters to initialize the EM algorithm. The model learning controls include the settings of the fixed number and the varying number of bins, as well as a threshold for fusing too close Gaussians, and a threshold for vanishing Gaussians with a too low number of values, as described in detail in the *g3mclass* user’s manual on https://g3mclass.readthedocs.org site. Suppose several histograms are used for the semi-constrained EM algorithm initialization. In that case, *g3mclass* automatically selects the mathematically preferred model with the lowest Bayesian information criterion (BIC) that measures the model's fit to the test data and outputs the test model parameters. The classification of references and queries relies on the selected GMM for the test. Individual models could be optimized for each analyte across the different study populations. The *g3mclass* additional functionalities include the output of spreadsheets with summary statistics (the mean, median, and standard deviation values for each class) and classification heatmaps.

The *g3mclass* performs consecutively three classification types—*proba, cutoff,* and *stringent cutoff (a.k.a. s.cutoff)* for a given test model to maximize the automated classification accuracy. The *g3mclass proba* utilizes the Bayesian approach to classify data. It classifies each value based on *a maximum a-posteriori* probability estimate of class membership. There is no a *priori* assumption about the number of classes. However, during this initial step, some analyte values in the test may be incorrectly assigned by the *proba* classification. This situation may occur when a component of GMM has a wide dispersion with its tails picking up values that otherwise belong to a different class (Fig. 2B). To directly address the separation of the GMM modes, *g3mclass* computes a set of cutoffs and autocorrects the potential *proba* misclassification. At *cutoff* classification step, data parsing is performed based on a minimal misclassification value with equal weights relative to adjacent classes. The consecutive *s.cutoff* classification relies on either the left or right interval values computed for a minimal misclassification cutoff that can be interpreted as tolerable intervals of the misclassification error rate (a tradeoff between misclassification of one type for misclassification of the other kind). During s*.cutoff* classification, more values are assigned to class 0 by the expansion of cutoff intervals. If the weight of class 0 in the test GMM is close to null, the *proba* classification of reference is invalid (Fig. 2C). However, *cutoff* and *s.cutoff* classification results may be valid for test and reference, depending on the degree of overlap for those samples. The lower overlap, the higher the accuracy of the *g3mclass* classification.

The *g3mclass* has a feature that allows users to evaluate parameter stability. The user can subsample the selected fraction from the original sample (reference, test, or both) up to 100 times. Resampling is done randomly without replacement to limit the risk of creating false classes based on repeated events from far-tailed distributions. The independent GMM is learned for each resample, and parameter estimates are provided. If, for example, a user selects the option of variable bin number, the software will apply it to each resample. The optimal number of classes for each resample will be based on BIC and may or may not differ from sample to sample. The user may compare the variability of the estimates of the biomarker classifier parameters: the number of classes, the mean values, and the diagnostic cutoffs separating the reference-like values from the disease-related test values (e.g., cutoffs between class 0 and class − 1; class 0 and class 1). Hence the user may base the judgment not only on the original model but also on the potential outcomes resulting from resampling. One way to conceptualize the utility of *g3mclass* in the field of biomarkers is to consider its value in updating the existing knowledge about biomarkers in reference samples (e.g., before disease or before treatment) and assessing the biomarker change in the test (e.g., in disease or post-treatment).

### Multiclass classification on a single analyte: biomarker or therapeutic target

**Figure 3:**
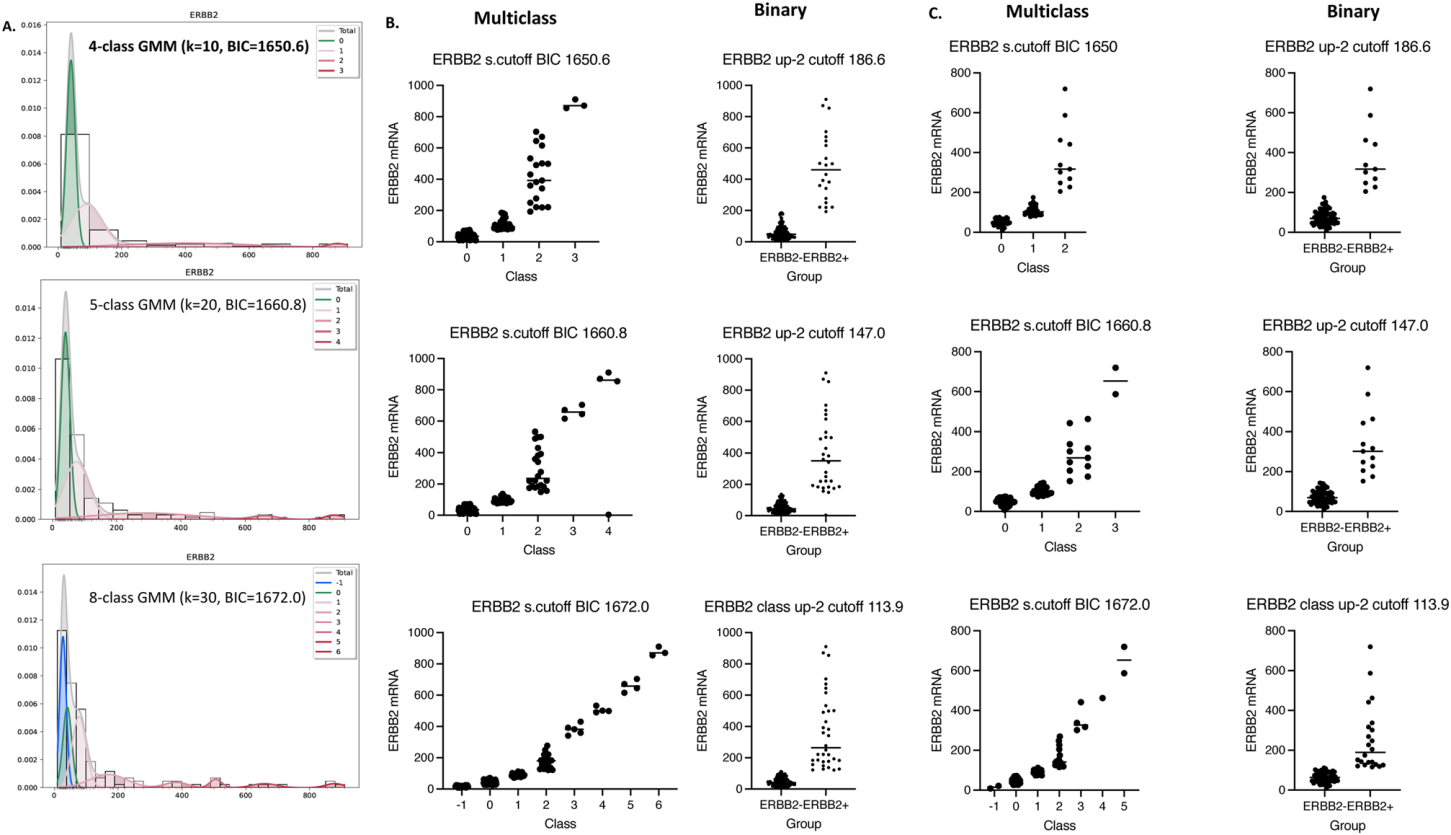
The *g3mclass*-assisted autoclassification on *ERBB2* mRNA. (**A**) A total mixture model and GMM's components for the test *ERBB2* mRNA was obtained with the *g3mclass*. The data distribution is shown as a PDF overlaid on a histogram and estimated by the means values, standard deviation, and weights of each component. The total GMM (gray); separate components are as follows: green—class 0 with the mean value of reference; class/es with the mean value lower than that of reference (blue) and class/es with the mean value higher than that of reference (red). The *ERBB2* mRNA model parameters including the mean ± standard deviation values and weights per class are: 4-class GMM: class 0 (40.4 ± 13.6; weight 0.46); class 1 (88.7 ± 48.9, weight 0.38), class 2 (403 ± 189, weight 0.14), class 3 (879 ± 29.1, weight 0.02); 5-class GMM: class 0 (40.4 ± 13.6; weight 0.43), class 1 (74.8 ± 37.9; weight 0.36), class 2 (287 ± 155; weight 0.17), class 3 (661 ± 38.7; weight 0.03), class 4 (878 ± 28.6; weight 0.02); 8-class GMM: class − 1 (27.0 ± 10.1; weight 0.21), class 0 (40.4 ± 13.6; weight 0.20), class 1 (77.2 ± 21.8; weight 0.28), class 2 (168 ± 57.6; weight 0.14), class 3 (381.0 ± 36.0; weight 0.04), class 4 (506 ± 18.6; weight 0.03); class 5 (659 ± 38.1; weight 0.03); class 6 (878 ± 28.6; weight 0.02). The best fit was obtained for the 4-class GMM based on the lowest BIC = 1650.6. (**B**) Graphs: Examples of the multiclass and subsequent binary classifications of IBC on *ERBB2* mRNA*.* (**C**) Graphs: Examples of multiclass and subsequent binary classification of DCIS on *ERBB2* mRNA.

First, we demonstrated the capabilities of *g3mclass* to automatically classify samples on the gene expression data for the established diagnostic/drug-response biomarker, the *ERBB2* encoding HER2. The mRNA levels were measured by a QuantiGene Plex 2.0 (QG2) assay in the intended-use population (as described in “Materials and methods”), where there is a regular need to differentiate among the patients with breast cancer. For the test and reference input, we used mRNA measurements from invasive breast carcinoma (IBC) and mammoplasties (noncancer), respectively. Additionally, we queried this gene expression data from the independent cohort of patients diagnosed with ductal carcinomas in situ (DCIS) on the pre-operative biopsy. To estimate the number and characteristics of cancer classes on *ERBB2* mRNA data, we used the *g3mclass* default parameters with varying bins (vector of k: 10, 15, 20, 25, 30, 35, 40). Among the models learned by *g3mclass* on varying histograms, the software picked GMM with the lowest BIC value = 1650.6 and the bin number k = 10, as illustrated in Fig. 3A. Based on the *g3mclass*-selected model, the *ERBB2* mRNA test values fit into the 4-class GMM. Class 0 is like a reference (in terms of the mean value and standard deviation); classes 1, 2, and 3 have increased mean values of *ERBB2* mRNA compared to a reference. The software learns new models by selecting a higher fixed number of bins in a histogram. For example, with k = 20 and BIC = 1660.8, the *ERBB2* mRNA test values fit into 5 class-GMM. Class 0 is like reference (as above); classes 1, 2, 3, and 4 have the increased mean value relative to the reference. This model allows a more detailed classification of *ERBB2* mRNA's test values and may be preferred in some clinical applications. With the increased number of bins, k = 30 and BIC = 1672.0, the *ERBB2* mRNA test values fit into 8-class GMM. This model provides an even more detailed classification; however, the model overfitting may occur, and reference may be represented by two classes − 1 and 0.

Considering all three models, we performed classification of the IBC, DCIS, and mammoplasty samples on *ERBB2* mRNA (Table 1). The *g3mclass* automatically stratified heterogeneous populations into multiple classes with differential levels of *ERBB2* expression, each of which was represented by Gaussian distribution. Apart from computing the proportions of each class as shown in Table 1, the *g3mclass* provides spreadsheet records on individual sample membership and summary statistics. For example, depending on the test GMM, an estimated 3–15% of *ERBB2* mRNA values from the reference data belonged to class 1, whereas the majority of *ERBB2* mRNA values from the test and query were classified into class 2 and higher that have not been present in the reference. Thus, it was reasonable to suggest that the software computed up-2 cutoff, which separated class 2 and higher from lower classes, was a diagnostic cutoff point associated with *ERBB2/HER2* overexpression in the subset of breast cancers.

**Table 1:** Summary of multiclass classification of breast tissue on *ERBB2* mRNA.

Sample	Model	Method	Class number							
			− 1	0	1	2	3	4	5	6
IBC	4-class GMM	proba		55.63%	29.58%	12.68%	2.11%			
		cutoff		55.63%	28.87%	13.38%	2.11%			
		s. cutoff		60.56%	23.94%	13.38%	2.11%			
DCIS		proba		36.00%	50.67%	13.33%				
		cutoff		34.67%	50.67%	14.67%				
		s. cutoff		49.33%	36.00%	14.67%				
Noncancer		proba		94.12%	5.88%					
		cutoff		94.12%	5.88%					
		s. cutoff		100.00%	00.00%					
IBC	5-class GMM	proba		54.23%	26.76%	14.08%	2.82%	2.11%		
		cutoff		52.11%	27.46%	15.49%	2.82%	2.11%		
		s. cutoff		59.86%	19.72%	15.49%	2.82%	2.11%		
DCIS		proba		30.67%	53.33%	14.67%	1.33%			
		cutoff		32.00%	50.67%	14.67%	2.67%			
		s. cutoff		48.00%	34.67%	14.67%	2.67%			
Noncancer		proba		91.18%	8.82%					
		cutoff		88.24%	11.76%					
		s. cutoff		97.06%	2.94%					
IBC	8-class GMM	proba	33.80%	14.79%	28.17%	11.97%	3.52%	2.82%	2.82%	2.11%
		cutoff	30.28%	19.72%	26.06%	12.68%	3.52%	2.82%	2.82%	2.11%
		s. cutoff	14.08%	45.07%	16.90%	12.68%	3.52%	2.82%	2.82%	2.11%
DCIS		proba	14.67%	17.33%	42.67%	17.33%	4.00%	1.33%	2.67%	
		cutoff	12.00%	20.00%	38.67%	20.00%	5.33%	1.33%	2.67%	
		s. cutoff	2.67%	42.67%	25.33%	20.00%	5.33%	1.33%	2.67%	
Noncancer		proba	47.06%	38.24%	14.71%					
		cutoff	38.24%	47.06%	14.71%					
		s. cutoff	5.88%	91.18%	2.94%					

### The diagnostic performance of the *g3mclass* modeling solutions

Going a step further, we examined how well a HER2 status may be predicted from *ERBB2* mRNA expression data using the *g3mclass* computed cutoffs because proteins are traditional therapeutic targets. We used the standardized statistical methods requiring biomarker dichotomization. For this purpose, we considered the current FDA-approved method for determining a binary HER2 status in breast cancer as a gold standard. We stratified HER2 positive (HER2+) and HER2 negative (HER2−) IBC based on the pathology reports. To test how well the measurements of *ERBB2* mRNA predict HER2+, we dichotomized the *ERBB2* mRNA data into groups using *g3mclass*-calculated cutoffs for three models. Choosing a model-predicted cutoff value, i.e., 186.6 (4-class GMM), 147.0 (5-class GMM), and 113.9 (8-class GMM), allows the transformation of multiclass into binary classification, at the same time eliminating equivocal results (Fig. 3B). When 186.6 is chosen as the *ERBB2* expression cutoff, the sensitivity is 70%, and the specificity is 99% (Table 2). When the cutoff is decreased to 147.0, the sensitivity is increased to 90%, and the specificity is reduced to 97%. When the cutoff is further reduced to 113.9, the sensitivity increases to 93%, while the specificity decreases to 94%. Thus, if binary classification on *ERBB2* mRNA is desired, cutoff up-2 may represent a tradeoff between sensitivity (the fraction of HER2+ cancers that are correctly identified by *ERBB2* mRNA assay as being HER2+) and specificity (the fraction of HER2− cancers that are correctly identified by *ERBB2* mRNA assay as not being HER2+). Considering the prevalence of HER2+ human breast cancer 20%, the estimated positive predictive values were 96%, 90%, 79%, and negative predictive values were 93%, 97%, 98%. The accuracy of the *ERBB2* mRNA test was 93%, 96%, 94% in 4-class, 5-class, and 8-class GMM, respectively.

**Table 2:** Diagnostic performance of *g3mclass* solutions for *ERBB2* mRNA test in IBC.

Model	4-class GMM (BIC 1650.6; Cutoff 186.6)	5-class GMM (BIC 1660.8; Cutoff 147.0)	8-class GMM (BIC 1672.0; Cutoff 113.9)
Statistic	Value (95% confidence interval)	Value (95% confidence interval)	Value (95% confidence interval)
Sensitivity	70.00% (50.60–85.27%)	89.66% (72.65–97.81%)	93.10% (77.23–99.15%)
Specificity	99.11% (95.13–99.98%)	97.35% (92.44–99.45%)	93.81% (87.65–97.47%)
Disease prevalence*	21.13% (14.73–28.77%)	20.42% (14.12–28.00%)	20.42% (14.12–28.00%)
Positive predictive value*	95.45% (74.64–99.34%)	89.66% (73.81–96.38%)	79.41% (65.15–88.84%)
Negative predictive value*	92.50% (87.71–95.52%)	97.35% (92.62–99.07%)	98.15% (93.29–99.51%)
Accuracy*	92.96% (87.43–96.57%)	95.77% (91.03–98.43%)	93.66% (88.31–97.06%)

*These values are dependent on HER2+ disease prevalence.

Additionally, using the *g3mclass* modeling solutions, we calculated the *ERBB2* mRNA diagnostic test parameters for query—an independent cohort of patients diagnosed with DCIS (Fig. 3C and Table 3). For 75 biopsies successfully profiled in QG2 assay, HER2 status by IHC could be assessed in 70 samples freshly cut from the same blocks of tissue that remain available after gene expression analysis. For the *g3mclass* selected 4-class GMM with the lowest BIC = 1650.6 and 186.6 as a cutoff, the accuracy, i.e., an overall probability that a patient's pre-surgical biopsy being correctly classified on a binary HER2 status from the *ERBB2* mRNA expression data, was 92.86% (95% confidence interval 84.11–97.64%), the positive predictive value was 100%, while the negative predictive value was 91.53% (95% confidence interval 84.11–97.64%). Thus, the results of *g3mclass* data analyses show the accuracy of identifying breast cancer potentially sensitive to anti-HER2 therapy and the robustness of the software in making the correct classification without equivocal results in the test and independent query.

**Table 3:** Diagnostic performance of *g3mclass* solutions for *ERBB2* mRNA test in DCIS.

Model	4-class GMM (BIC 1650.6; cutoff 186.6)	5-class GMM (BIC 1660.8; cutoff 147.0)	8-class GMM (BIC 1672.0; cutoff 113.9)
Statistic	Value (95% confidence interval)	Value (95% confidence interval)	Value (95% confidence interval)
Sensitivity	68.75% (41.34–88.98%)	68.75% (41.34–88.98%	68.75% (41.34–88.98%)
Specificity	100.00% (93.40–100.00%)	98.15% (90.11–99.95%)	83.33% (70.71–92.08%)
Disease prevalence*	22.86% (13.67–34.45%)	22.86% (13.67–34.45%)	22.86% (13.67–34.45%)
Positive predictive value*	100.00%	91.67% (60.55–98.75%)	55.00% (38.20–70.73%)
Negative predictive value*	91.53% (83.93–95.72%)	91.38% (83.66–95.64%)	90.00% (81.16–94.95%)
Accuracy*	92.86% (84.11–97.64%)	91.43% (82.27–96.79%)	80.00% (68.73–88.61%)

*These values are dependent on HER2+ disease prevalence.

### Multiclass classification on multiple biomarkers

**Figure 4:**
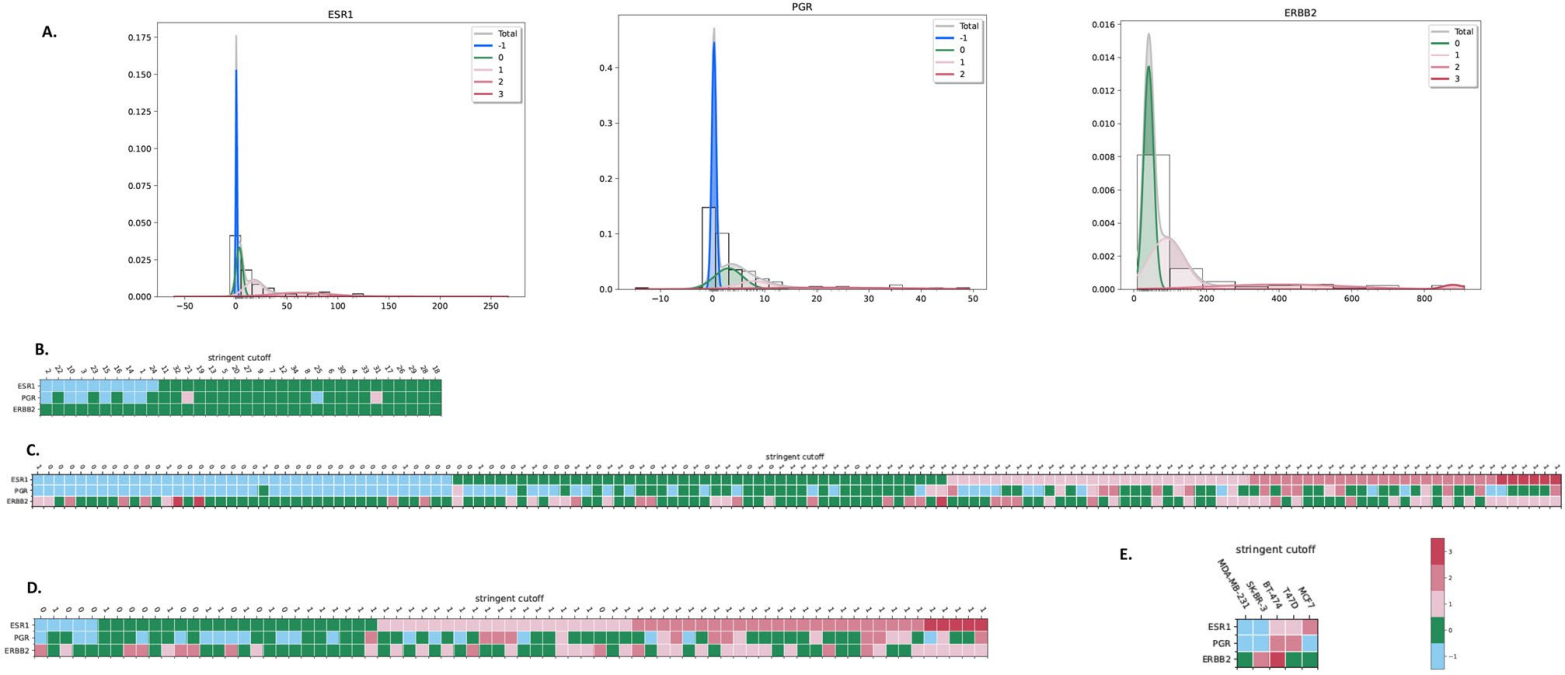
The *g3mclass* autoclassification on three genes. (**A**) The *g3mclass*-selected models. The 5-class model parameters for *ESR1* mRNA: class − 1 (0.49 ± 0.57; weight 0.25); class 0 (3.34 ± 3.18; weight 0.27), class 1 (17.4 ± 9.22; weight 0.25), class 2 (63.4 ± 29.2; weight 0.19), class 3 (99.5 ± 105; weight 0.05). The 4-class model parameters for *PGR* mRNA: class − 1 (0.30 ± 0.43; weight 0.48); class 0 (3.09 ± 2.77; weight 0.26), class 1 (7.88 ± 3.49; weight 0.13), class 2 (63.4 ± 29.2; weight 0.12). The 4-class model parameters for *ERBB2* mRNA as above in Fig. 2. (**B**)–(**E**) The *g3mclass*-created classification heatmaps. The *s.cutoff* classification heatmaps on *ESR1, PGR,* and *ERBB2* for mammoplasties (**B**), IBC (**C**), DCIS (**D**), and IBC cell lines (**E**). The IHC score for ER-positive (1), and ER-negative (0) cancers are on the top of heatmaps (**C**, **D**). In E, human breast cancer cell lines: MDA-MB-231 (triple-negative), SK-BR-3 (HER2 overexpressing), BT-474 (luminal B), T47D (luminal A), MCF7 (luminal A). Bar, heatmap’s color scale for classes.

The *g3mclass* can easily be upscaled to analyze data from multiplexing assays. The data processing steps are the same as those for a single biomarker, i.e., preparation and entry of input data, modeling, classification, and analysis of the output results. We ran *g3mclass* to concurrently classify breast tissue samples on *ERBB2, ESR1,* and *PGR* mRNA measurements obtained in the validated and highly reliable multiplex QG2 assay (“Materials and methods”). These target genes encode HER2 and human steroid hormone receptors—estrogen receptor alpha (ER), and progesterone receptor (PR), abnormal presence of which defines treatments, such as anti-HER2 and hormonal therapies^21^. We obtained clinical markers' binary status (positive vs. negative) from pathology reports (“Materials and methods”). We input the mRNA expression data from the test (IBC), reference (mammoplasties), and two queries (DCIS and five human breast cancer cell lines) as one file into *g3mclass.* Instantaneously, the software selected and depicted each gene's mathematically favorable test model (Fig. 4A). It also performed three sequential classifications (*proba, cutoff,* and *s.cutoff*) for each model. Finally, it summarized data into spreadsheets and heatmaps. Using the resampling feature of the *g3mclass*, we found that diagnostic cutoff estimates for *ESR1* were stable despite the appearance/disappearance of far-tailed classes (Supplementary Table 1). To illustrate the essence of the tumor's classification on *ESR1* in the context of the other two genes, we present heatmaps built for *s.cutoff* classification that improves the specificity (Fig. 4B–E).

As depicted in Fig. 4B, we found two groups of noncancerous breast tissues—with no activity of *ESR1* (class − 1) and with physiological levels of the *ESR1* transcript (class 0) based on a 5-class test GMM for *ESR1* mRNA. In cancer, three other groups emerged with either slightly (class 1), moderately (class 2), or highly increased (class 3) levels of *ESR1* mRNA (Fig. 4C, D). In our study populations, the *ESR1’s* transcript levels were abnormally increased in 40% of IBC, 64% of DCIS, and 0% of noncancer, based on the up-1 cutoff separating class 0 from 1 and higher. The *ERBB2* mRNA was abnormally high in 16% of IBC, 15% of DCIS, and 0% of noncancer, considering up-2 as a diagnostic cutoff. Thereby *g3mclass* automatically selected tumors potentially sensitive to endocrine and anti-HER2 therapy, while other cancers may need different types of treatments. Notably, the *g3mclass* estimates showed that about 24% of IBC and 19% of DCIS, scored as ER-positive in pathology reports had reference-like levels of *ESR1* transcript. These cases are candidates for overdiagnosis. The potential underdiagnosis was estimated in 0% of IBC and 1% of DCIS. Concurrently, the *g3mclass* provided insights into the variability of *PGR*, encoding steroid hormone receptor PR, a marker recommended for testing in IBC but not in DCIS^22^. High levels of *PGR* mRNA (class 2) were found in 9% of IBC, 13% of DCIS, and 0% of noncancer. In sharp contrast, low/undetectable levels of *PGR* mRNA (class − 1) were in 48% of IBC, 25% of DCIS, and 21% of reference. Thus, the *g3mclass* revealed low/or loss of *PGR* mRNA expression in IBC and the upregulation of *PGR* mRNA in DCIS in our study populations. Finally, we queried an independent set of mRNA data from the human breast cancer cell lines with the know expression levels of HER2, ER/PR^23,24^ and found them classified according to the established status (Fig. 4E).

In short, we demonstrated the diagnostic accuracy of the *g3mclass* analysis of clinical biomarkers and therapeutic targets. Additionally, we showed the robustness of this software in the automated multiclass classification of the test and independent queries. We have also provided evidence of the scalability of the *g3mclass* software to classify and visualize classifications on multiple analytes concurrently. We demonstrated the software output's interpretability by showing how various valuable insights can be extracted from raw test and query data using the *g3mclass*. More importantly, we showed how the *g3mclass* helps analyze each person's cancer with a unique pattern of biomarkers.

## Discussion

Modern biomedical science requires highly specialized but easy-to-adapt software. This article presents *g3mclass*, a practical stand-alone application for a general biomedicine task concerning molecular assay data classification. The *g3mclass* offers inference about the number of classes, the mean and spread levels of an analyte in each class, and the prevalence of each class in the study population. In oncology, this allows unraveling and taking full advantage of hidden unique patterns of biomarkers and targets in each person's cancer. In addition, it may help researchers in the early stages of pharmaceutical testing of new therapies and companion diagnostics to determine whether further, often expensive, studies are warranted.

In the present article, we demonstrated how *g3mclass*-assisted classification helps human experts quickly assess the biological variability of gene transcripts across the populations of women diagnosed with primary breast cancer without extensive and long-term data collection. We also provided how human experts may select among probabilistic GMMs automatically learned by the *g3mclass* software. GMM is often used for unsupervised clustering, mainly for data exploration. An example of such an approach is subgrouping cancers based on the similarity of gene expression patterns^25–27^. The *g3mclass* exploits the customized semi-constrained EM algorithm's ability to learn test models from known (provided by experts) and unknown (missing values) information. This computational approach is the opposite of supervised classifications requiring the predefined knowledge of the number of the mixture components. For example, a two-component mixture model sorts differentially expressed genes in microarray experiments^12^. The principal innovation of *g3mclass* is embedding pre-existing experts’ knowledge of reference parameters into the test GMM. As a result, it substantially improves the differentiation of new-to-test versus reference-like values and provides biological and clinical context for interpretation outcomes. This approach defines the significant difference of *g3mclass* from other powerful software packages handling Gaussian finite mixture modeling as their clustering capabilities, including the most popular R package mclust^14^ and the Addinsoft XLSTAT (https://www.xlstat.com/en/company). Overall, there are two critical applications of the *g3mclass* in the biomedical field. First, it enables the discovery of previously unknown groups with different levels of biomarkers, including those that are not part of the reference and thus are more likely linked with disease. Second, it allows individual patient classification in line with personalized clinical decision-making.

This article focused primarily on validated biomarkers because the quality of HER2 and ER diagnostics affects millions worldwide. According to World Health Organization, with an estimated 2.3 million new annual cases reported globally, female breast cancer is the most diagnosed cancer type (https://www.who.int/news-room/fact-sheets/detail/breast-cancer). Experts recommend that every primary IBC be tested for the presence of HER2 and ER and re-tested in subsequent recurrences and metastases by semi-quantitative immunohistochemistry (IHC) and/or fluorescence in situ hybridization (FISH)^22,28^. These tests may produce equivocal results that could not be interpreted as positive or negative. The challenge remains to define either at the protein^8,29^ or the mRNA^30–33^ the HER2/ER expression cutoffs that segregate patients who may derive meaningful clinical benefit from endocrine and targeted therapies from those who will not. We have previously demonstrated that dichotomization of structurally mixed mRNA data with a single cutoff, e.g., using a ROC model or two-component GMM, may result in the loss of reliable information about the patient groups, as well as misclassification of some individuals^19^. This article presented practical statistical software to help remedy such a problem and demonstrated how multiclass classification with *g3mclass* may help fine-tune stratification on clinical biomarkers. In our study cohorts, *g3mclass* automatically recognized cancers unlikely to be present in the reference, i.e., *ERBB2* mRNA + (class 2 and higher) and *ESR1* mRNA + (class 1 and higher). Likewise, recognizing by *g3mclass* the group of *ERBB2* mRNA + (class 1) may help define HER2-low positive breast cancer in clinical trials^34^. Clinical studies adopting *g3mclass* are warranted to investigate whether the groups with differentially increased levels of *ERBB2* mRNA and *ESR1* mRNA have different sensitivity to the targeted therapy.

In clinical trial designs, *g3mclass* provides experts with a flexible diagnostic cutoff driven by the intended use where the sensitivity or specificity is more beneficial. HER2 and ER are the targets of the emerging therapies for breast cancer and other types of cancer^35,36^. The cutoffs necessary for testing the clinical benefits of new therapies are likely to differ across cancer types^37–39^. Using *g3mclass,* experts may tailor the biomarker cutoff for each disease. If a more standard binary classification is desired, experts may choose a data-driven approach and transform multiclass into binary classification while eliminating equivocal results. To further unravel the *g3mclass* capabilities in determining the biological and clinical value of candidate biomarkers, we provide evidence of the scalability of the *g3mclass* software to classify on multiple analytes and visualize concurrent classifications with heatmaps. This approach may help manage ER+/HER2+ cancers, as the dual implementation of the hormone and anti-HER2 therapies showed evidence of success in the clinical trials^8^.

In sum, the applicability of *g3mclass* may be easily extended beyond one biomarker, dataset, or disease. It provides a cost-effective and straightforward way to examine and deal with the variability of the molecular assay data. The analysis with the *g3mclass* does not depend on the computing environment, which ensures the research's reproducibility. In clinical settings, applying *g3mclass* promises more precise stratification that may help improve therapeutic sensitivity. Yet, the limitations for the *g3mclass* application exist, as outlined in this article and software documentation. To what degree this molecular classifier combined with the genomic classifier and conventional clinicopathological characteristics improves patient outcomes remains to be seen in clinical studies. The free dissemination of *g3mclass* paves the path towards such investigational studies and a personalized treatment approach.

## Materials and methods

### Human tissues and cell lines

The formalin-fixed paraffin-embedded (FFPE) human breast tissues were obtained from the Department of Pathology and Laboratory Medicine, Tumor Tissue and Biospecimen Bank, and the Cooperative Human Tissue Network at the University of Pennsylvania. The study was performed with 256 samples, including 34 mammoplasties from women with no history of breast cancer, 75 diagnostic biopsies of ductal carcinomas in situ (DCIS), 142 surgical excisions of primary invasive breast cancer (IBC), and 5 human breast cancer cell lines. The cell lines MCF-7T-47D, MDA-MB-231, SK-BR-3, and BT-474 were purchased from the American Type Culture Collection and cultured accordingly. MycoAlert Assay (Cambrex) confirmed that mycoplasma-free cells were used in the experiments. The tissue samples were accrued randomly from the same geographic region. Summaries of the characteristics of the study populations have been published^19,40^.

### Direct messenger RNA (mRNA) profiling

For mRNA data collection, we ran QuantiGene Plex 2.0 (QG2) assay (Genospectra/Panomics/Affymetrix/eBioscience/ThermoFisher Scientific, USA) and read on FlexMap 3D (Luminex/Merck Millipore) according to manufacturers’ protocol and as described in detail^19^. QG2 is a highly reliable and validated molecular assay that uses amplified branch DNA (bDNA) technology for parallel gene expression profiling^41,42^. We analyzed measurements of specific probes with the QG2 assay kits for quantitation of multiple target specific RNAs directly in lysates from FFPE tissue and cell lines. Our human Plex Set 12988 included 14 target-specific and two housekeeping gene probes described in detail^40^. Here, we analyzed mRNA for *ERBB2* (probe set region 1203–1621),* ESR1* (probe set region 5671–6292), and *PGR* (probe set region 2609–3194)*.*

### Clinical markers

The status of steroid hormone receptors (ER and PR) and HER2 were determined by IHC and/or FISH, the FDA-approved methods. The status of ER, PR for all tumors, and HER2 for primary IBC were obtained from surgical pathology reports. In addition, HER2 status in DCIS was assessed based on the FDA-approved method for IBC as described^40^.

### Statistical analyses and modeling

In this article, modeling and analyses of datasets were performed with *g3mclass* v.1.2 on macOS Mojave v.10.14.6. with Python v.3.9.5 and wxPython v. 4.1.1. Additionally, we used MedCalc, a diagnostic test evaluation calculator at https://www.medcalc.org/calc/diagnostic_test.php (Version 20.027; accessed March 2, 2022) and GraphPad Prizm 9.3.0 (GraphPad Software, LLC, La Jolla, CA, USA) to visualize group comparisons.

### Ethics approval

Studies were conducted in accordance with recognized ethical guidelines. We used approval from the University of Pennsylvania Institutional Review Board committee with a waiver of written informed consent to analyze patients’ tissue and records.

## Supplementary Information

Supplementary Information 1.

Supplementary Information 2.

## Data Availability

All data needed to evaluate the article's conclusions are present in the article or the Supplementary Information. Series record GSE214540 provides access to QuantiGene Plex 2.0 16-gene expression data submitted to the GEO repository. In addition, the *g3mclass* is available as a standalone application on https://pypi.org/project/g3mclass site.

## Code availability

The *g3mclass* software is written in Python, and its graphical user interface (GUI) is based on *wxPython*. Its documentation is distributed with the software but also is available online https://g3mclass.readthedocs.io. It includes installation instructions as well as a step-by-step workflow example. The *g3mclass* was tested on macOS, Windows, and Linux platforms but should run on any other platform offering Python3 and wxPython. It is distributed under OpenSource license GPL-2, freely installable from https://pypi.org/project/g3mclass with the standard Python *pip* tool. The code source is freely accessible from https://github.com/MathsCell/g3mclass.
