# Supplementary material for: The g3mclass is a practical software for multiclass classification on biomarkers
Source: Sci Rep. 2022 Nov 5;12:18742. doi: 10.1038/s41598-022-23438-9 (PMC9637185; doi:10.1038/s41598-022-23438-9)
Supplement: Supplementary file 1 — Supplementary Information 1. [file 41598_2022_23438_MOESM1_ESM.docx]

| **Biomarker** |  |  | **GMM class** | | | | | | | | **Diagnostic cutoff** | | | |
| --- | --- | --- | --- | --- | --- | --- | --- | --- | --- | --- | --- | --- | --- | --- |
| ***ESR1*** |  |  | class -2 | class -1 | class 0 | class 1 | class 2 | class 3 | class 4 | class 5 | down_left | down-1 | up-1 | up_right |
| **TEST SAMPLE** |  | **mean** |  | **0.486** | **3.34** | **17.4** | **63.4** | **99.5** |  |  | **1.191** | **1.585** | **8.843** | **11.511** |
|  |  | SD |  | 0.567 | 3.18 | 9.22 | 29.2 | 105 |  |  |  |  |  |  |
| **REF RESAMPLE** |  |  |  |  |  |  |  |  |  |  |  |  |  |  |
| fraction | 0.9 | **mean** |  | **0.483** | **3.29** | **17.3** | **63.3** | **99.6** |  |  | **1.18** | **1.57** | **8.73** | **11.3** |
| number | 5 | std |  | 0.00859 | 0.223 | 0.346 | 0.413 | 0.0709 |  |  | 0.034 | 0.0424 | 0.355 | 0.473 |
|  |  |  |  |  |  |  |  |  |  |  |  |  |  |  |
| fraction | 0.9 | **mean** |  | **0.485** | **3.33** | **17.4** | **63.4** | **99.6** |  |  | **1.19** | **1.58** | **8.81** | **11.5** |
| number | 10 | std |  | 0.00755 | 0.175 | 0.281 | 0.324 | 0.0621 |  |  | 0.0285 | 0.0355 | 0.296 | 0.411 |
|  |  |  |  |  |  |  |  |  |  |  |  |  |  |  |
| fraction | 0.9 | **mean** |  | **0.486** | **3.32** | **17.4** | **63.5** | **99.6** |  |  | **1.19** | **1.58** | **8.82** | **11.5** |
| number | 100 | std |  | 0.007 | 0.166 | 0.254 | 0.287 | 0.0527 |  |  | 0.0258 | 0.0322 | 0.267 | 0.371 |
|  |  |  |  |  |  |  |  |  |  |  |  |  |  |  |
| fraction | 0.75 | **mean** |  | **0.486** | **3.37** | **17.4** | **63.4** | **99.6** |  |  | **1.19** | **1.59** | **8.85** | **11.5** |
| number | 100 | std |  | 0.0129 | 0.312 | 0.488 | 0.562 | 0.061 |  |  | 0.0481 | 0.06 | 0.495 | 0.683 |
|  |  |  |  |  |  |  |  |  |  |  |  |  |  |  |
| fraction | 0.5 | **mean** |  | **0.486** | **3.38** | **17.3** | **63.3** | **99.5** |  |  | **1.19** | **1.59** | **8.82** | **11.5** |
| number | 100 | std |  | 0.02 | 0.579 | 0.838 | 1.09 | 0.239 |  |  | 0.0799 | 0.0997 | 0.824 | 1.09 |
| **REF & TEST RESAMPLE** |  |  |  |  |  |  |  |  |  |  |  |  |  |  |
| fraction | 0.9 | **mean** |  | **0.456** | **3.37** | **29.6** | **70.9** |  |  |  | **1.15** | **1.52** | **9.82** | **12.3** |
| number | 5 | std |  | 0.0335 | 0.225 | 14.6 | 4.28 |  |  |  | 0.0626 | 0.0883 | 1.03 | 0.918 |
|  |  |  |  |  |  |  |  |  |  |  |  |  |  |  |
| fraction | 0.9 | **mean** |  | **0.473** | **3.37** | **27** | **71.3** |  |  |  | **1.18** | **1.55** | **9.69** | **12.2** |
| number | 10 | std |  | 0.0322 | 0.195 | 13 | 3.73 |  |  |  | 0.0515 | 0.0756 | 1 | 0.907 |
|  |  |  |  |  |  |  |  |  |  |  |  |  |  |  |
| fraction | 0.9 | **mean** |  | **0.48** | **3.35** | **23.6** | **68.4** | **127** |  |  | **1.18** | **1.56** | **9.36** | **11.9** |
| number | 100 | std |  | 0.0564 | 0.178 | 11.3 | 5.42 | 39.7 |  |  | 0.0845 | 0.119 | 0.976 | 0.888 |
|  |  |  |  |  |  |  |  |  |  |  |  |  |  |  |
| fraction | 0.75 | **mean** |  | **0.492** | **3.34** | **28.1** | **70.7** | **140** | **204** |  | **1.27** | **1.71** | **10.1** | **18.7** |
| number | 100 | std |  | 0.0794 | 0.319 | 13.3 | 13.3 | 46.2 |  |  | 0.307 | 0.477 | 2.85 | 22.3 |
|  |  |  |  |  |  |  |  |  |  |  |  |  |  |  |
| fraction | 0.5 | **mean** | **-29.5** | **0.539** | **3.42** | **27.5** | **70.8** | **84.5** | **96.4** | **81** | **1.27** | **1.71** | **10.1** | **18.7** |
| number | 100 | std |  | 0.237 | 0.525 | 14.5 | 31.9 | 31.2 | 21.1 |  | 0.307 | 0.477 | 2.85 | 22.3 |

**Supplementary Table 1.** **Re-evaluating stability of model parameters for *ESR1* mRNA test.** The mixture and diagnostic cutoff parameters were reassessed using the g3mclass feature for resampling reference (ref) or reference and test together (ref & test). The mean values of diagnostic cutoffs between class 0 and class – 1 (down-1; down_left); class 0 and class 1 (up-1; up-right) appeared stable. The larger the resample fraction (>75%), the smaller the variance of the parameters and fewer far-tailed distributions. Fraction is the proportion of the original sample that has undergone resampling. Number, the number of resamples. SD, the standard deviation for class means; std, the standard deviation for resampled means and cutoffs.

**Supplementary Data.** **ESR1, PGR, ERBB2_ER code IBC_DCIS_BC cells.txt.**

The ***g3mclass*** analyzed QG2 assay data for *ESR1, PGR,* and *ERBB2* expression is organized in columns. Left to right:

id (ref), *ESR1* (ref), *PGR* (ref), and *ERBB2* (ref) for mammoplasty samples (n=34);

id (test), *ESR1* (test), *PGR* (test), and *ERBB2* (test) for IBC samples (n=142);

id (DCIS), *ESR1* (DCIS), *PGR* (DCIS), and *ERBB2* (DCIS) for the queried DCIS samples (n=75);

id (BC cells), *ESR1* (BC cells), *PGR* (BC cells), and *ERBB2* (BC cells) for the queried human breast cancer cell lines (n=5).
